# Under nutrition, maternal anemia and household food insecurity are risk factors of anemia among preschool aged children in Menz Gera Midir district, Eastern Amhara, Ethiopia: a community based cross-sectional study

**DOI:** 10.1186/s12889-019-7293-0

**Published:** 2019-07-19

**Authors:** Getabalew Engidaye, Mulugeta Melku, Aregawi Yalew, Zegeye Getaneh, Fikir Asrie, Bamlaku Enawgaw

**Affiliations:** 1 Amhara Regional State Debre Berhan Health Science College, Debre Berhan, Ethiopia; 2 Department of Hematology & Immunohematology, School of Biomedical and Laboratory Sciences, College of Medicine and Health Sciences, University of Gondar, Gondar, Ethiopia

**Keywords:** Anemia, Pre-school aged children, Menz Gera Midir, Ethiopia

## Abstract

**Background:**

In Ethiopian, the prevalence of anemia among preschool aged children widely varied across regions. Since anemia adversely affects the cognitive and physical development of the children, it is important to determine its burden for implementing appropriate measurements. Therefore, this study was aimed at determining the anemia prevalence and associated factors among preschool aged children.

**Method:**

A community based cross-sectional study was conducted on a total of 432 preschool children in Menz Gera Midir district from January to May, 2017. A multi stage sampling procedure was applied to select the target groups. Hemocue analyzer for Haemoglobin determination; anthropometric measurements for assessment nutritional status, structured questionnaires for socio-demographic and economic variables were used for data collection. The morphological appearance of red blood cell was assessed microscopically to determine type of anemia. Descriptive statistics were employed to summarize the data and binary logistic regression was used for inferential statistics. A *p* value less than 0.05 was considered as statistically significant.

**Result:**

The overall prevalence of anemia was 123 (28.5%); of which 38 (30.9%) and 85 (69.1%) were moderate and mild, respectively. Morphologically about 50.4, 37.4 and 12.2% were microcytic hypochromic, normocytic normochromic and macrocytic anemias, respectively. Child age 6-11 months (COR: 5.67, 95% CI: 2.2, 14.86), child age 12–23 months (COR: 5.8, 95% CI: 2.3, 14.7), wasting (COR: 3.5, 95% CI: 1.2, 9.8), stunting (COR: 3.8, 95% CI: 1.92, 7.77), underweight (COR: 2.12, 95% CI: 1.07, 4.38), MUAC measurement below 13 cm (COR: 5.6, 95% CI: 2.83, 11.15), household headed by female (COR: 3.24, 95% CI: 1.1, 9.63), maternal anemia (COR: 4, 95% CI: 2.2, 7.23) and household food insecurity (COR: 2.12, 95% CI: 1.09, 4.12) were significantly associated with anemia.

**Conclusion:**

The prevalence of anemia among the children was found to be high and associated with child age group, child nutritional status, house hold headed by female, maternal anemia and household food insecurity. Further studies on nutritional anemia, community based nutritional education, iron supplementation to children at risk should be promoted.

## Background

Anemia is a condition where hemoglobin concentration is decreased in the blood. It is a serous public health problem worldwide that affects both wealthy and poor countries with a significant adverse health consequences as well as adverse impacts on social and economic development [1–3]. Anemia is considered as severe, moderate, mild and no public health problem, if its prevalence is ≥40%, 20–39.9%, 5–19.9 and < 5%, respectivel in the community [1].

According to the 2008 WHO report, 56.3% of world’s preschool aged children reside in developing countries including sub-Saharan Africa where anemia is a severe public health problem [4–6]. Golobally, the 2015 WHO report showed, 42.6% anemia prevalence among children aged 6–59 months in 2011. The WHO Africa, South East Asia and Eastern Mediterranean regions account the highest prevalence. These three regions account 62.3, 53.8 and 48.6% anemia prevalence, respectively. The report also revaled that 50% anemia prevalence among children aged 6–59 months in Ethiopia [1]. Evidences also revealing that its prevalence showed a rising in children compared to other age groups [7–9]. Since the demand of iron is higher in preschool children for rapid growth, anemia prevalence is higher. It affects children both physically and mentally. As a result, working capacity and educational performance of children will be decreased [2, 4, 10, 11].

According to the Ethiopia Demographic and Health Survey (EDHS) reports of 2005, 2011 and 2016, the national prevalence of anemia was estimated to 53.5%,44.2 and 56% in preschool aged children, respectively and its prevalence in Amhara regional state was 54, 35.1 and 41%, respectively [7, 12, 13]. In Ethiopian, its prevalence among preschool aged children widely varied across regions that have been ranged from 33% (Addis Ababa) to 75.2% (Afar region) and disparity of prevalence also observed between urban and rural children, which is higher in rural children [7]. Usually, this high prevalence of anemia is associated with food insecurity resulted from floods and drought [14]. These disasters are common problem in Ethiopia including the study area, Menz Gera Midir district. The floods and drought causes rugged terrain and degraded lands which causes a decline in agricultural land productivity [15]. This inturn cuases food insecurity and micronutrient deficiency anemia [3, 16, 17]. Therefore, determining the prevalence and associated risk factors of anemia have paramount importance for policy makers in order to ensure sustainable improvements. But in the study area, which is suffer from food insecurity with an incidence of 84.7% [14], there is limited information regarding the anemia prevalence and its predictive factors. Thus, this study was aimed to assess the prevalence and predictive factors of anema among pre-school aged children in the Menz Gera Midir district, Eastern Amhara, North Shewa zone, Ethiopia.

## Methods

### Study setting and population

A community based cross-sectional study was conducted on a total of 432 preschool children in Menz Gera Midir district from January to May, 2017. The district is located in Semien Shewa Zone, Eastern Amhara state, Ethiopia. The administrative center of this district is Mehal Meda town which is located 295 km away from Addis Ababa and 165 km from its zonal town (Debre Birhan). The town is also elevated 3037 m above sea level with latitude/longitude of 10^0^18′17″ N/39^0^ 39′ 31″E. According to the population projection of Ethiopia from 2014 to 2017, this district has a total population of 138,708 with 16,361(11.8%) urban inhabitants [18]. Children aged 6–59 months reside in the selected kebeles for at least 6 months and whose parents/guardians are willing to fully participate in the study were included. While, children with active hemorrhage, history of blood transfusion within the last 2 months, history of surgery within the last 2 months were excluded from the study.

### Sample size determination

To determine the required sample size for study, a single population proportion formula was used as denoted below

$$ \mathbf{n}=\frac{{\boldsymbol{Z}}^2\boldsymbol{p}\left(1-\boldsymbol{p}\right)}{{\boldsymbol{d}}^2}=\frac{(1.96)^2\ast 0.25\left(1-0.25\right)}{(0.05)^2}=\mathbf{288} $$

Where z = Z score for 95% confidence interval, which is 1.96

*p* = expected prevalence of anemia, which was 25% taken from Menz Keya [19].

d = tolerable error between the sample and true population, which is 5%

Considering affordable resources for investigations, a design effect of 1.5 for sampling error was taken and 288*1.5 = 432 children were included.

### Data collection procedures

**Fig. 1:**
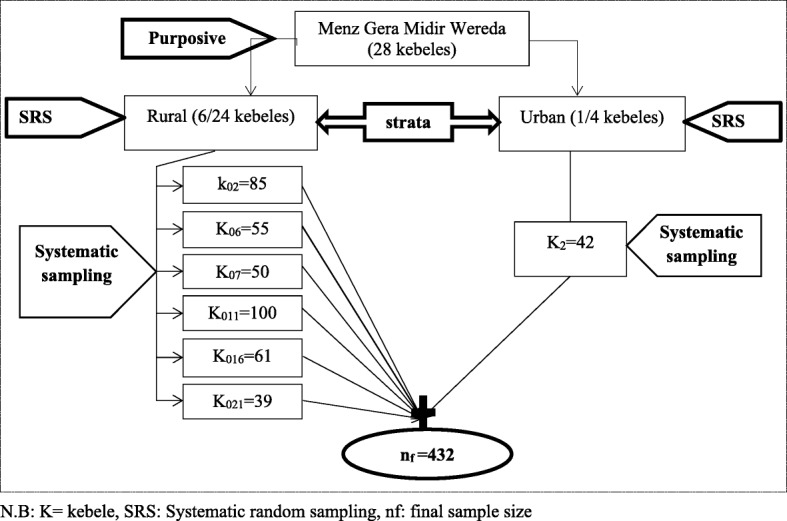
Schematic representation of sampling procedure. N.B: K = kebele, SRS: Systematic random sampling, nf: final sample size

A multi-stage sampling procedure was used; at the first stage, sample was determined to collect from one fourth of the total kebeles; out of 28 kebeles, seven kebeles (1 urban and 6 rural) were selected randomly. Kebele (neighbourhood) is the smallest administrative unit in Ethiopia. At the second stage, the number of households included from each kebeles were proportionally allocated. Then a systematic sampling method was used to select each household (Fig. 1). The total numbers of households in each kebele was taken from each administrative kebele and used to calculate the sampling interval (K) which was 26. After the first household randomly selected, households every 26th interval were approached. If a household was with two or more eligible childern, only one of them was chosen randomly by lottery method. On the otherhand, when the selected household was closed even after revisit or child was not eligible, the next household was included.

### Household demographic and socio-economic data collection

A pretested structured questionnaire which was prepared based on the national survey questionnaire and accordingly modified based on the reviewed literature [20] was used. The questionnaire consisted of socio-demographic characteristics of the child and their parents, household food security status (HFSS), child feeding practices, food consumption pattern and health condition of the children. Household food security (HHFS) status was assessed by using the standardized questionnaire developed by Food and Nutritional Technical Assistance (FANTA) [21, 22]. Food consumption pattern and dietary diversity scores (DDS) were determined by using a modified Helen Keller International Food Frequency Questionnaire (FFQ) and a 24-h dietary recall, respectively [22–24]. Altitude for each kebele was measured using an accurate altimeter app android installed on smart phone.

### Wealth index determination

Wealth index was determined to assess inequalities in household characteristics, in the use of health and other services. It used as an indicator of level of household wealth. The index was determined using household assets and type of house. A principal component analysis (PCA) was used to produce a common factor score for each household. Variables were coded between 0 and 1, entered and analyzed. Then variables with communality values of > 0.5 were used to produce factor scores. Factor scores were summed and categorized into three relative measures of socio economic status of households as low, medium and rich [22].

### Household food insecurity assessment

The Food and Nutritional Technical Assistance (FANTA) scale guideline questions were used to assess household food security status. The questionnaire was adapted from household food insecurity access scale and validated for developing countries. A household was considered as food secured if it had experience of less than the first 2 food insecurity indicators from the 27. Each question was responded as never, rarely, sometimes, or often. Households were considered as “food secure,” if they “never” or “rarely” worried about the deficiency of food in their households [21, 25].

### Dietary assessment

Food frequency was assessed with a questionnaire consisted of ten groups of food items was used. The food items were grouped in to cereals, legumes, meat, egg, vegetables, fruits, dairy products, fish and sea foods, sweet foods made with sugar, honey, oil, fat, or butter and any other foods, such as condiments, coffee, tea. Then dietary diversity scores (DDS) was calculated from these food groups and categorized as high (DDS ≥ 7), medium (DDS = 4–6), and low (DDS ≤ 3) [22].

### Anthropometric measurements

Child age, weight, height and mid-upper arm circumference (MUAC) were measured according to the 2008 WHO recommendation for nutritional status assessment [26]. All Childrens’ weight was measured while wearing light-weight cloth with a portable weight scale to the nearest 0.1 kg. The weighing scale was calibrated using the standard calibration weight of 2 kg iron bars. Child height was measusered using a locally manufactured wooden standiometre with a sliding head bar to the nearest 0.1 cm in Frankfurt position (head, shoulder, buttocks, knee, and heals touch the vertical board). Children height aged below 24 months was taken while lying down and for those older children heights were measured at standing position. Measurements of weight and height were taken twice and the average was recorded. Then the data were entered into WHO Anthro 3.2.2.1 for the calculation of weight-for-age (WAZ), weight-for-height (WHZ) and height-for-age (HAZ) standard Z-scores. Children were classified as stunted, underweight and wasted when HAZ, WAZ and WHZ scores were < −2SD, respectively [27].

### Hemoglobin (Hgb) measurement

Haemoglobin values of children and their mothers was determined by using HemoCue Hb 300^+^ analyzer*.* Since the study area altitude is > 3000 m above sea level, results of Hgb were adjusted to its respective sea level by subtracting 1.9 g/dL as it is recommended by WHO [28]. Anemia was defined when Hgb level is < 11 g/dL for both genders. Regarding to anemia severity Hgb value of 10 to 10.9 g/dL, 7 to 9.9 g/dL and < 7 g/dL were considered as mild, moderate and severe anemia, respectively [28].

### Peripheral blood examination

The morphological characteristics of RBC were assessed by using 100x (oil immersion) high magnification power light microscope. The type of anemia then was classified based on the characteristics of RBC morphology as microcytic, normocytic and macrocytic anemias.

### Stool examinations

For intestinal parasite examination, stool samples were collected from study participants and wet mount was prepared by using normal saline for direct microscopy. The remaining portion of the collected sample was preserved by using 10% formalin andconcentration techniques. Finally, examination of stool by using concentration technique for ova or parasite was performed within 24 h of collection.

### Statistical analysis methods

Firstly, data were checked for completeness and coded manually. After coding, data were double entered and stored using EPI-info 7 and exported to SPSS 20 for further analysis. Descriptive statistics were used to summarize the characteristics of the study population. To determine factors associated with anemia, bivariate logistic regression analyses was done and the 95% confidence (CI) level was used determined the strength of association between the predictors and dependent variables. Those variables with a *P* value of < 0.2 in bivariate analysis were fitted in to multivariate logistic regression analysis. A *P* value < 0.05 in multivariable analysis was considered as statistically significant.

## Results

### Socio-demographic characteristics of children

A total of 432 preschool aged children, 390 (90.3%) from rural and 42 (9.7%) from urban were included. Of them, 227/390 (58.2%) and 27/42 (64.3%) were females from rural and urban residence, respectively. The median age of the children was 24 months with interquartile range (IQR) of 14–42 months. From 432 children, 49 (11.3%), 217 (50.2%) and 121 (28%) were wasted, stunted and underweight, respectively. During the study period, stool sample was collected from 251 children. From them about 41 (16.3%) were positive for one of the following intestinal parasites; *Entamoeba histolytica*, *Giardia lamblia*, *Ascaris Lumbricoides*, *Hookworm* and *Taenia saginata.* Of those children infected by intestinal parasites, 35 (85.4%) were found anemic (Table 1).

**Table 1:** Characteristics of the preschool aged children

Background characteristic	Frequency	Percentage
Sex of child		
Male	178	41.2
Female	254	58.8
Age Group (in months)		
6–11	90	16.7
12–23	107	25.0
24–35	70	17.1
36–47	76	18.8
48–59	89	22.5
Delivery Status		
Term	425	98.4
Preterm	7	1.6
History of illness in the past 2 weeks		
No	415	96.1
Yes	17	3.9
Place of Child Birth		
Health Facility	297	68.8
Home	136	31.2
Intestinal parasites (*n* = 251)		
Positive	41	16.3
Negative	210	83.7
Vaccination Status		
No vaccinated	5	1.2
Partially Vaccinated	58	13.4
Fully Vaccinated	369	85.4
Wasting		
Yes	49	11.3
No	383	88.7
Stunting		
Yes	217	50.2
No	215	49.8
Underweight		
Yes	121	28.0
No	311	72.0
MUAC		
≥ 13.5 cm	167	38.7
< 13.5 cm	265	61.3

### Parental socio-demographic and economic characteristics

About 420 (97.2%) of the care givers were female and 414 (95.8%) of them were mothers in relationship to the child. Majority, 402 (93%), of the mother respondents were married. Regarding paternal educational status, more than one third of 158 (36.6%) of the mothers cannot read and write, and 411 (95.2%) of them were housewives. The median household size in rural and urban was 5 (IQR: 4–6) and 4 (IQR: 3–5) person per household respectively. Economically, nearly half of the households categorized under lower socio-economic class. Around 72.9% of the households were suffering from food insecurity (Table 2).

**Table 2:** Parents’ socio- demographic and economic characteristics

Background characteristic	Frequency	Percentage
Sex of the child care giver		
Female	420	97.2
Male	12	2.8
Residence		
Rural	390	90.3
Urban	42	9.7
Relationship with care giver		
Mother	414	95.8
Father	12	2.8
Others	6	1.4
Marital Status		
Married	402	93.0
Divorced	25	5.8
Single	5	1.2
Sex of the head of the household		
Male	406	93.99
Female	26	6.01
Occupation of Mother		
Housewife	411	95.2
Government employed	14	3.2
Merchant	7	1.6
Father’s Occupation		
Farmer	365	84.5
Governmental Employee	22	5.1
Labor	19	4.4
Merchant	26	6.0
Maternal Educational status		
No Education	158	36.6
Primary School	155	35.9
Secondary completed	98	22.7
Higher Education Completed	21	4.9
Father’s Educational status		
No Education	94	21.8
Primary School	191	44.2
Secondary completed	125	28.9
Higher Education complete	22	5.1
Household Wealth quintile		
Higher	64	14.8
Medium	164	38.0
Lower	204	47.2
Household dietary Diversity Score (HDDS)		
Low	188	43.5
Medium	214	49.5
High	30	7.0
Family size in the household		
≤ 3	98	22.7
4–6	275	63.7
≥ 7	59	13.6
Household Food Security		
Food Secured	117	27.1
Food Insecured	315	72.9

### Prevalence of anemia

**Fig. 2:**
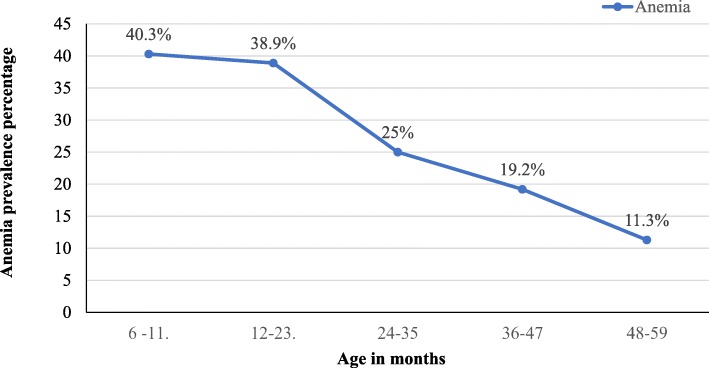
Anemia distribution among preschool aged children by their age

The mean (± SD) value of Hgb level was 11.3 ± 1.1 g/dL with values ranging from 7.3 g/dL to 14.8 g/dL. The overall prevalence of anemia was 123 (28.5%) (95% CI: 24.2–32.7) with 116 (29.7%) and 7 (16.7%) magnitude among rural and urban children, respectively. Of the 123 anemic children, 38 (30.9%) and 85 (69.1%) were moderately and mildly anemic, respectively. Morphologically, about 50.4, 37.4 and 12.2% of anemic cases were microcytic-hypochromic, normocytic-normochromic and were macrocytic, respectively. There was no significant difference in the prevalence of anemia between females and males which was 29.5 and 27.0%, respectively. The prevalence of anemia among wasted, stunted, underweight and MUAC children was 67.3, 36.4, 40.5 and 40%, respectively. The prevalence of anemia was higher among younger children (40.3%) in (6–11 months), and it had shown decline as the child’s age increased (Fig. 2).

### Factors associated with anemia

Table 3 shows bivariate and multivariate analysis of factors associated with anemia. In bivariate analysis, child and maternal related variables such as child age group, vaccination status of the child, wasting, stunting, underweight, MUAC, sex of the household head, maternal education, maternal anemia and household food insecurity showed association with anemia. Then those variables having *p*-value less than 0.2 were subjected to multivariable binary logistic regression. In multivariable analysis of factors associated with anemia, child age 6–11 months (COR: 5.67, 95% CI: 2.2, 14.86) and 12–23 months (COR: 5.8, 95% CI: 2.3, 14.7), wasting (COR: 3.5, 95% CI: 1.2, 9.8), stunting (COR: 3.8, 95% CI: 1.92, 7.77), underweight (COR: 2.12, 95% CI: 1.07, 4.38), MUAC measurement < 13 cm (COR: 5.6, 95% CI: 2.83, 11.15), household headed by female (COR: 3.24, 95% CI: 1.1, 9.63), maternal anemia (COR: 4, 95% CI: 2.2, 7.23) and household food insecurity (COR: 2.12, 95% CI: 1.09, 4.12) were significantly associated with anemia. On the other hand child sex, residence, vaccination status, intestinal parasite, maternal age, maternal occupation, delivery status, history of illness with in last 2 weeks and place of birth did not show any statistically significant association with anemia.

**Table 3:** Association of characteristics of study subjects with anemia

Characterstics of study subjects	Anemic	Non-anemic	COR (95% CI)	AOR (95%CI)
Sex of child				
Female	75 (29.5)	179 (70.5)	1	
Male	48 (27.9)	130 (73.0)	0.881 (0.58–1.35)	
Child age group (months)				
6–11	47 (52.2)	43 (47.8)	5.27 (2.41–11.55)	5.67 (2.20–14.86)^a^
12–23	33 (30.8)	74 (69.2)	4.98 (2.40–10.40)	5.80 (2.30–14.7)^a^
24–35	21 (30.0)	49 (70)	2.61 (1.14–5.93)	2.14 (0.78–5.84)
36–47	16 (21.1)	60 (78.9)	2.99 (1.36–6.61)	1.71 (0.22–4.367)
48–59	6 (6.7)	83 (93.3)	1	1
Residence				
Urban	7 (16.7)	35 (83.3)	1	1
Rural	116 (29.7)	274 (70.3)	2.17 (0.91–4.90)	1.833 (0.524–6.407)
Delivery status				
Preterm	3 (42.9)	4 (57.1)	1.91 (0.33–10.240	
Term	120 (28.2)	305 (71.8)	1	
History of illness in the past 2 weeks				
Yes	5 (29.4)	12 (70.0)	1.05 (0.362–3.04)	
No	118 (28.4)	297 (71.6)	1	
Place of Birth				
Home	35 (25.9)	100 (74.1)	0.83 (0.52–1.32)	
Health Facility	88 (29.6)	209 (70.4)	1	
Intestinal Parasites (*n* = 251)				
Positive	35 (85.4)	6 (14.6)	0.55 (0.218–1.38)	
Negative	160 (76.2)	50 (23.8)	1	
Vaccination Status				
Not Complete	26 (41.3)	37 (58.7)	1.97 (1.13–3.42)	0.682 (0.294–1.584)
Complete	97 (26.3)	272 (73.7)	1	1
Wasted				
Yes	33 (67.3)	16 (32.7)	6.72 (3.53–12.76)	3.5 (1.20–9.8)^a^
No	90 (23.5)	293 (76.5)	1	1
Stunted				
Yes	79 (36.4)	138 (63.8)	2.23 (1.45–3.42)	3.8 (1.92–7.77)^a^
No	44 (20.5)	171 (79.5)	1	1
Under Weight				
Yes	49 (40.5)	72 (59.5)	2.18 (1.40–3.41)	2.12 (1.07–4.38)^a^
No	74 (23.8)	237 (76.2)	1	1
MUAC				
≥13.5 cm	17 (10.2)	150 (89.8)	1	1
< 13.5 cm	106 (40.0)	159 (60.0	5.88 (3.37–10.28)	5.6 (2.83–11.15)^a^
Maternal Age				
< 30 years	91 (30.7)	205 (69.3)	1.44 (0.91–2.30)	1.043 (0.504–2.155)
> = 30 Years	32 (23.5)	104 (76.5)	1	1
Sex of the Household head				
Female	8 (66.7)	4 (33.3)	5.3 (1.57–17.9)	3.24 (1.10–9.63)^a^
Male	115 (27.4)	305 (72.6)	1	1
Maternal Education				
No Education	53 (33.5)	105 (66.5)	4.80 (1.08–21-36)	1.16 (0.897–72.53)
Primary School	39 (25.2)	116 (74.8)	3.20 (0.71–14.34)	5.85 (0.759–45.07)
Secondary School	29 (29.6)	69 (70.4)	3.9 (0.87–18-26)	7.21 (0.898–57.86)
Higher Education	2 (9.6)	19 (90.4)	1	1
Maternal Anemia				
Yes	56 (56.6)	43 (43.4)	5.64 (3.46–9.22)	4.0 (2.20–7.23)^a^
No	57 (18.8)	247 (81.2)	1	1
Household food insecurity				
Secured	23 (19.7)	94 (80.3)	1	
Insecured	100 (31.7)	215 (68.3)	1.90 (1.13–3.18)	2.12 (1.09–4.12)^a^
Household dietry diversity score (HDDS)				
Low	62 (33)	126 (67)	1.97 (0.76–5.06)	
Medium	55 (25.7)	159 (74.3)	1.38 (0.54–3.56)	
High	6 (20)	24 (80)	1	

^a^indicated statistically significant variables

## Discussion

The findings of this study showed 28.5% overall prevalence of anemia, denoting a moderate public health problem according to WHO classification [1]. This result was in agreement with a study conducted in the neighboring district, Menz Keya and higher than Menz Lalomma [19]. On the other hand the observed prevalence was lower when compared to the national and regional prevalence reported by EDHS 2005, 2011 and 2016 [7, 12, 13]. The possible reasons for inconsistencies in the prevalence of anemia across studies might be related to variation of study time in which the studies were conducted, geographical variability of risk factors, differences in socioeconomic status of the populations and implementation of different strategies to minimize the burden of anemia in the region where these studies have been conducted.

The study showed that most of the children had mild type of anemia 85 (69.1%) and only 38 (30.9%) had moderate anemia. There was no severe anemia which was as expected because it is a community based study, not a hospital based. This is supported by a study conducted in Northwestern Uganda [29]. There was no significant difference in the prevalence of anemia between females and males which was 29.5 and 27.0%, respectively. Half (50.4%) of the type of anemia was microcytic-hypochromic anemia followed by normocytic-normochromic (37.4%) and macrocytic anemias (12.2%). This might be due the fact that the community in the study area consume cereals which have low iron content; thus, microcytic hypochromic anemia is dominantly observed type of anemia. On the other hand, vitamin B12 and/or folate deficiency may cause the development of macrocytic anemia.

From the study result, age was remained significantly associated with anemia and this finding has been reported in several other studies conducted in northern Ethiopia [5, 30], Ghana [31] and Nigeria [32]. As the child getting older, the prevalence of anemia decreased from 40.5% (in the age group of 6–11 months) to 11.3% (in the age group of 48–59 moths) which meant that children at younger age were the most vulnerable group for anemia. The likelihood of being anemic for the children in the age group of 6–11 and 12–23 months was about six fold greater as compared to children in the age group of 48–59 months.

The greatest prevalence of anemia in the age group of 6–11 and 12–23 months might be attributable to different factors. This is may be due to the transition from feast to famine of iron store due to the rapid growth and expanding of blood volume during the first 2 years. As studies conducted in India [10], Nigeria [32] and Sub-Saharan Africa [4] showed, children within this age group require large amount of iron to maintain a near steady mean hemoglobin concentration within their body. Since the iron stores in their body has usually been depleted and exclusive breast feeding might not contain adequate iron, the initiation of complementary foods with high iron content is mandatory to satisfy their high iron demand. Even though complementary diet is a crucial source of iron during this time, the major source of diet in the study area was cereal groups which have low iron content [33]. In addition to this, after the age of 24 months, they use more diversified diet which in turn increases the quality and quantity of foods. Moreover, the amount of iron required per body kilogram relatively decreases due to the slowdown of growth rate as the child gets older [2].

Maternal anemia during the time of survey was another factor that statistically associated with childhood anemia. The odds of anemia for children whose mother was anemic was about four times higher as compared to children whose mothers were non-anemic. This association has also been reported by studies conducted in Cameroon [34], Northern Ethiopia [5] and Eastern Cuba [9]. The possible reason for this association might be that both mothers and children mostly share a common home environment, which involves mutual exposure to a common set of physical, socioeconomic, and dietary conditions. Furthermore, maternal anemia might be associated with poor birth outcomes such as low birth weight and prematurity of the child. Thus, hemoglobin level of the child is strongly dependent on the level of maternal hemoglobin concentration in their blood. In addition to this, maternal anemia might led to limited fetal iron store and the amount of iron secreted by the breast milk might be insufficient for daily iron requirement of the child. Therefore, due to these consequences of maternal anemia, the possibility of childhood anemia is greater in children whose mothers were anemic. Thus, it is important to screen pregnant women for anemia and regular nutrient supplementation during their follow-ups.

Wasting, stunting and underweight were significantly associated with anemia in preschool aged children. This association was supported by a studies conducted in Kilte Awlalo zone Northern Ethiopia [35], Tigray Province [5], Nigeria [32], United Republic of Tanzania [36] and Pakistan [37]. Children who were wasted, stunted, underweight and whose MUAC measurement below 13 cm were 3.5, 3.8, 2.12 and 5.6 times more likely to have anemia compared to children with who were not undernourished, respectively. Stunting is an indicator of chronic malnutrition whereas underweight is a combination of both chronic and acute malnutrition. Usually, anemia and undernutrition often share common causes because both these factors are aggravated by poverty and food insecurity. Undernourished children are more suffered from inadequate bioavailability of micronutrients such as iron, B12 and folate in their body which are important for the formation of blood cells. Therefore, those children who are undernourished cannot form adequate blood cells as many as required; consequently the this leads to the development of nutritional deficiency anemia which is common especially in developing countries [38].

Household food insecurity was also identified as an associated factor for childhood anemia which implies children from food insecured household were 2.34 times greater at risk of developing anemia. This finding is supported by similar studies conducted in Tigray province and rural Cameroon [36]. From this study nearly three forth (72.9%) of the household were food insecured which is almost similar with previous data conducted in the area [7, 14]. The higher prevalence of anemia in those children living in food insecured households might be due to inadequate intake of diets, consumption of a limited variety of food groups with low iron content and coping mechanism during food shortage [21, 29]. The coping mechanisms during food shortage were factor that might be attributed to poor nutritional status, and thus for the development of anemia. During food shortage, the household planned to limit the portion of food size at meal time and reducing frequency of meals per day, feeding only working family members, and consuming less preferred or inexpensive food were the common coping mechanisms in the area to prevent early used up of reserved foods.

Another finding in this study was that, anemia was significantly associated with living in a household headed by females. Children from female headed household were 3.24 times at greater risk for anemia. This association can be explained by the time allocated for child care and household food security level. Most of the involvements to improve the child health and nutritional status rely on the behavior of their caregiver, often the mother. When females become the head of the household with no husband in the household, every task in and outside the house were on the shoulder of the women. In these circumstances, women focus on outside home activities which are both time and labor intensive tasks. The time allocated to care their children in the house is minimized. Therefore, initiation of complementary foods, close follow up of their children and timely breast feeding had been compromised. Consequently, the child become more vulnerable either for chronic or acute malnutrition which leads to the development of nutritional anemia [39].

As a strength, this study is a community based and tried to assess different factors of anemia among preschool aged children in rural part of eastern Amhara, northeast Ethiopia. The findings could be used as a base line information to conduct large scale study that determine the cause and effect relationship. There were also some limitations to this study ought to be taken into account when interpreting the results of this study. The first major limitation is being cross-sectional nature that does not allow us to observe causality in the relationship between anemia and its associated factors. Secondly, we were also unable to measure serum concentration of micronutrients to characterize anemia.

## Conclusion

The prevalence of anemia among preschool aged children in Menz Gera Midir district was a moderate public health problem. As shown by the study, children younger than 24 months of age were more likely to be affected by anemia than older age groups. Mild.and microcytic-hypochromic anemia were the dominant type of anemias observed. Nutritional status of the child (wasting, stunting, under weight and MUAC < 13 cm), maternal anemia, household food insecurity and female headed households were the contributing factors for the development of childhood anemia in the district. From the finding, it is recommended that community based nutritional education, nutritional support programs for children living in households with food insecured should be implemented to reduce the burden of anemia in the community. Households, those in food insecure status should be identified and included in safety net programs. Children living in those households should be included in school feeding programs. In addition, maternal hemoglobin. Finaly We recommend further studies that include larger sample size and assessment of micronutrients to characterize as iron, B12 and folate deficiency anemia should be conducted.

## Data Availability

The datasets used and/or analyzed during the current study available from the corresponding author on reasonable request.

## Abbreviations

AOR: Adjusted odds ratio
COR: Crude odds ratio
DDS: Dietary diversity scores
EDHS: Ethiopian demographic and health survey
FANTA: Food and Nutritional Technical Assistance
FFQ: Food Frequency Questionnaire
HAZ: Height for Age Z score
HFSS: Household food security status
Hgb: Hemoglobin
MUAC: Mid-upper arm circumference
PCA: Principal component analysis
RBC: Red blood cells
RDT: Rapid diagnostic test
WAZ: Weigh for Age Z score
WHO: World Health Organization
WHZ: Weigh for Height Z score
